# Supplementary material for: Digital healthcare interventions to support parents with acutely ill children at home: A systematic review
Source: PLOS Digit Health. 2025 Sep 15;4(9):e0000998. doi: 10.1371/journal.pdig.0000998 (PMC12435699; doi:10.1371/journal.pdig.0000998)
Supplement: S1 Table — (DOCX) [file pdig.0000998.s001.docx]

**S1 Table Exclusion table: digital interventions review**

|  |  | **Inclusion Criteria (x = criteria not met)** | | | | | | |
| --- | --- | --- | --- | --- | --- | --- | --- | --- |
|  |  | Research articles – Primary research or systematic review | Published in English 2014 onwards | Intervention – evaluation of digital intervention or digital component | Intervention – improve health literacy | Intervention- aimed at the prevention/treatment of acute child illness | Children aged under 19 years | Parents/caregivers responsible for children |
| Author, Year | **Title** |  |  |  |  |  |  |  |
| No author, 2015. | Resources parents turn to when their child is unwell.  Nursing Children & Young People, 27(1): 13. | X | Y | X | Y | Y | Y | Y |
| Aronson, P.L., Politi, M.C., Schaeffer, P., Fleisher, E., Shapiro, E.E., Niccolai, L.M., Alpern, E.R., Bernstein, S.L., Fraenkel, L. and Cloutier, R., 2021. | Development of an App to facilitate communication and shared decision-making with parents of febrile infants ≤ 60 days old.  Academic Emergency Medicine, 28(1): 46-59. | Y | Y | Y | X | X | Y | Y |
| Cabey, W.V., Shea, J.A., Kangovi, S., Kennedy, D., Onwuzulike, C. and Fein, J., 2018. | Understanding pediatric caretakers’ views on obtaining medical care for low-acuity illness.  Academic Emergency Medicine, 25(9): 1004-1013 | Y | Y | X | X | Y | Y | Y |
| Chew, A., A. Koshy and E. Lim, 2019. | "Prescribing health information - the development and evaluation of a smartphone app providing guidance and management advice for a feverish child." Future healthcare journal **6**: 90. | X  (published abstract only) | Y | Y | Y | Y | Y | Y |
| Chirambo GB, Thompson M, Hardy V, Ide N, Hwang PH, Dharmayat K, Mastellos N, Heavin C, O'Connor Y, Muula AS, Andersson B, Carlsson S, Tran T, Hsieh JC, Lee HY, Fitzpatrick A, Joseph Wu TS, O'Donoghue J., 2021. | Effectiveness of Smartphone-Based Community Case Management on the Urgent Referral, Reconsultation, and Hospitalization of Children Aged Under 5 Years in Malawi: Cluster- Randomized, Stepped-Wedge Trial. | Y | Y | Y | Y | Y | Y | X |
| Chiswell, E., Hampton, D., Okoli, C.T.C., 2019. | Effect of patient and provider education on antibiotic overuse for respiratory tract infections.  Journal for Healthcare Quality: Promoting Excellence in Healthcare, 41(3): e13-e20 | Y | Y | X | Y | X | Y | X |
| Costa Filho RV, Souza JN, Andrade LOM, Oliveira AMB, Denis JL, Ribeiro LLS, Ribeiro KG, Andrade DB, Pereira SSL., 2021. | Field of practice: lariiSa: smart digital solutions to support decision-making in Family Health Strategy management. | Y | Y | Y | Y | N | Y | N |
| da Penha JC, do Nascimento LA, de Sabino LMM, da Rocha Mendes ER, da Rocha SS, Roubert ESC, Lima FET, de Oliveira Melo RC, de Almeida PC, Melo ESJ, Barbosa LP., 2022. | Effects of Educational Interventions on Maternal Self‑efficacy and Childhood Diarrhea: A Randomized Clinical Trial. | Y | Y | N | Y | Y | Y | Y |
| De Vos-Kerkhof, E., Geurts, D. H.F., Moll, H.A., Oostenbrink, R., Wiggers, M., 2016. | Tools for ‘safety netting’ in common paediatric illnesses: a systematic review in emergency care.  Archives of Disease in Childhood, 101(2): 131-139. | Y | X (papers reviewed all published before 2014). | X | Y | Y | Y | Y |
| Donovan, E., Wilcox, C.R., Patel, S., Hay, A.D., Little, P., Willcox, M.L., 2020. | Digital interventions for parents of acutely ill children and their treatment-seeking behaviour: a systematic review.  The British Journal of General Practice: The Journal of the Royal College of General Practitioners, 70(692): e172-e178 | Y | Y/X (2/3 papers reviewed published before 2014. Remaining paper included in our review) | Y | Y | Y/X | Y | Y/X |
| Edwards, C., Bolling-Walker, K., Deupree, J., 2020. | Actionability and usability of a fever management tool for pediatric caregivers.  Journal of Continuing Education in Nursing, 51(7): 338-344 | X | Y | X | Y | Y | Y | Y |
| Gwiasda M, Schwarz S, Büssing A, Jenetzky E, Krafft H, Hamideh Kerdar S, Rathjens L, Boehm K, Martin D., 2022. | Changing knowledge and attitudes about childhood fever: testing a video instruction before its application in a health app. | Y | Y | X | Y | Y | Y | Y |
| Hart, L., R. Nedadur, J. Reardon, N. Sirizzotti, C. Poonai, K. Speechley, J. Loftus, M. Miller, M. Salvadori and N. Poonai, 2015. | "An interactive web-based module versus website and standard of care for parental fever education: A randomized controlled trial." Paediatrics and Child Health **20**(5): e90. | X  (published abstract only) | Y | Y | Y | Y | Y | Y |
| Jandasek BN, Kopel SJ, Esteban CA, Rudders SA, Spitalnick JS, Larsen MA, Cushman GK, McQuaid EL., 2024. | Friends, Family, and Food: Development of a Food Allergy Intervention, F3-App, for Children. | Y | Y | Y | Y | N | Y | N |
| Knight, K.M., Trehane, S.J., Chingono, J., Crossley, B., Cleugh, F., 2017. | Chip: child health information for parents-animated health education videos for the waiting area.  Archives of Diseases in Childhood, 102: A112 | X  (published abstract only) | Y | Y | Y | Y | Y | Y |
| Lepore, N., Hart, L., Poonai, N., 2017. | Web-based tools for educating caregivers about childhood fever: a randomized controlled trial.  European Journal of Emergency Medicine, 24(6): e28. | X  (published abstract only) | Y | Y | Y | Y/X | Y | Y |
| Luthy, K. E., A. Anderson, J. Macintosh, Beckstr, R. L. , L. M. Eden, R. Amy and C. I. Macintosh .2017. | "A WHOOPING COUGH EDUCATION MODULE for WIC Clients in Utah." MCN: The American Journal of Maternal Child Nursing **42**(5): 283-288. | Y | Y | Y | Y | X | Y | X |
| Mahmood, H., McKinstry, B., Luz, S., Fairhurst, K., Nasim, S., Hazir, T., 2020. | Community health worker-based mobile health (mHealth) approaches for improving management and caregiver knowledge of common childhood infections: a systematic review.  Journal of Global Health, 10(2): 020438 | Y | Y | Y | Y | Y | Y | N |
| Martin, D., Wachtmeister, J., Ludwigs, K., Jenetzky, E., 2020. | The FeverApp registry- ecological momentary assessment (EMA) of fever management in families regarding conformity to up-to-date recommendations.  BMC Medical Informatics and Decision Making, 20(1) | Y | Y | X | Y | Y | Y | Y |
| Mier, M., Antoon, J.W., Sefcovic, S., Awatramani, S., Kreppel, A., Smith, S.B., 2020. | Affecting length of stay in well-appearing febrile infants.  Pediatric Quality & Safety, 5(6): e359 | Y | Y | X | X | Y | Y | X |
| Miller, A.C., Singh, I., Koehler, E., Polgreen, P.M., 2018. | A smartphone-driven thermometer application for real-time population- and individual-level influenza surveillance.  Clinical Infections Diseases, 67(3): 388-397 | Y | Y | X | X | X | X | X |
| Mohammed, A., Franke, K., Okyere, P.B., Brinkel, J., Marinovic, A.B., Kreuels, B., Krumkamp, R., Fobil, J., May, J., Owusu-Dabo, E, 2017. | Feasibility of electronic health information and surveillance system (eHISS) for disease symptoms monitoring: a case of rural Ghana.  Plos One, 13(5) | Y | Y | X | Y | Y | Y | Y |
| Mohanan, M., Babiarz, K.S., Goldhaber-Fiebert, J.D., Miller, G., Vera-Hernandez, M., 2016. | Effect of a large-scale social franchising and telemedicine program on childhood diarrhea and pneumonia outcomes in India.  Health Affairs, 35(10): 1800-1809 | Y | Y | X | Y | Y | Y | X |
| Mohanan, M., Giardili, S., Das, V., Rabin, T.L., Raj, S.S., Schwartz, J.I., Seth, A., Goldhaber-Fiebert, J.D., Miller, G., Vera-Hernandez, M., 2017. | Evaluation of a social franchising and telemedicine programme and the care provided for childhood diarrhoea and pneumonia, Bihar, India.  Bulletin of the World Health Organization, 95(5): 343-352E | Y | Y | X | Y | Y | Y | X |
| Nair, H., Williams, L.J., Marsh, A., Lele, P., Bhattacharjee, T., Chavan, U., Hirve, S., Campbell, H., Juvekar, S., 2018. | Assessing the reactivity to mobile phone and repeated surveys on reported care-seeking for common childhood illnesses in rural India.  Journal of Global Health, 8(2) | Y | Y | X | X | Y | Y | Y |
| Oliphant, N., Manda, S. Daniels, K., Odendaal, W.A., Beseda, D., Kinney, M., Johansson, E.W., Doherty, T., 2021. | Integrated community case management of childhood illness in low-and middle-income countries.  Cochrane Database of Systematic Review, 2 | Y | Y | X | X | Y | Y | Y |
| Peetom, K.K.B., Smits, J.J.M., Ploum, L.J.L., Verbakel, J.Y., Dinant, G.J., Cals, J.W.L, 2016. | Does well-childcare education improve consultations and medication management for childhood fever and common infections? A systematic review.  Archives of Disease in Childhood, 102(3): 261-267 | Y | Y | Y | Y | Y | Y | X |
| Peetom, K.K.B, Crutzen, R., Verhoeven, R., Bohnen, J., Winkens, B., Dinant, G.J., Cals, J.W.L., 2018. | Optimizing decision-making among childcare staff on fever and common infections: cluster randomized controlled trial.  European Journal of Public Health, 29(3): 505-511 | Y | Y | X | Y | Y | Y | Y |
| Raval, B., Arpan, P., Ravindra, H.N. check names, 2015. | A study to assess the effectiveness of structured teaching programme on knowledge regarding “acute respiratory tract infection among mothers of under five children at Piparia, Vadodara.”  International Journal of Nursing Education, 7(3): 51-55 | Y | Y | X | Y | Y | Y | Y |
| Sabino LMM, Ferreira ÁMV, Mendes ERR, Melo ESJ, Penha JC, Lima KF, Nascimento LAD, Lima FET, Melo RC, de Almeida PC, Barbosa LP., 2021. | Educational interventions using a primer and motivational interviewing: randomized clinical trial. | Y | Y | N | Y | Y | Y | Y |
| Sanchez, D., Reiner, J.F., Sadlon, R., Price, O.A., Long, Michael, W., 2019. | Systematic review of school telehealth evaluations.  Journal of School Nursing, 35(1): 61-76 | Y | Y | Y | X | Y | X | X |
| Schmidt M, Babcock L, Kurowski BG, Cassedy A, Sidol C, Wade SL., 2022. | Usage Patterns of an mHealth Symptom Monitoring App Among Adolescents with Acute Mild Traumatic Brain Injuries. | Y | Y | Y | Y | Y | Y | N |
| Shimony-Kanat S, Gofin R, Nator N, Solt I, Abu Ahmad W, Liebergall Wischnitzer M, Lawen H, Kopitman A, Crassac V, Kerem E., 2023. | Mothers’ Knowledge of Infants' Fever Management: A National Prospective Study. | Y | Y | N | Y | Y | Y | Y |
| Siew, L., Hsiao, A., McCarthy, P., Agarwal, A., Lee, E., Lei, C., 2016. | Reliability of telemedicine in the assessment of seriously ill children.  Pediatrics, 137(3): 49 | Y | Y | Y | X | Y | Y | X |
| Sivakumar, A., Venkatramanan, P., Premkumar, S., 2020. | Role of the internet in the health-seeking behaviour of parents of children under-five during fever.  Indian Journal of Public Health Research and Development, 11(3): 482-484 | Y | Y | X | Y | Y | Y | Y |
| Sun, V., 2017. | The effects of fever management education on increased use of fever reducing measures among parents.  Academic project | Unable to source the paper |  |  |  |  |  |  |
| Tavan A, Monemi E, Keshavarz F, Kazemi B, Nematollahi M., 2022. | The effect of simulation-based education on parental management of fever in children: a quasi-experimental study. | Y | Y | N | Y | Y | Y | Y |
| Wilkinson, A., 2017. | Prehospital assessment of a child under one year with fever.  Emergency Nurse, 24(10): 28-33 | X | Y | X | Y | Y | Y | Y |
| Zakaria, N.A., Saleh, F.N.B.M., Razak, M.A.A., 2018. | Internet of things based infant body temperature monitoring. Conference Paper : 148-153. | X | Y | X | X | Y | X | X |
| Tutar Güven Ş, İşler Dalgiç A, Duman Ö., 2020 | Evaluation of the efficiency of the web-based epilepsy education program (WEEP) for youth with epilepsy and parents: A randomized controlled trial. | Y | Y | Y | Y | Y | Y | Y |
