# Supplementary material for: Digital healthcare interventions to support parents with acutely ill children at home: A systematic review
Source: PLOS Digit Health. 2025 Sep 15;4(9):e0000998. doi: 10.1371/journal.pdig.0000998 (PMC12435699; doi:10.1371/journal.pdig.0000998)
Supplement: Attachment — Submitted filename: Response to Reviewers.docx [file pdig.0000998.s004.docx]

**Response to authors comments:**

Thank you for taking the time to review the manuscript and offer feedback. We have responded to each comment individually as outlined below. Do let us know if you require further information.

Journal Requirements:

1. Please upload separate figure files in .tif or .eps format. Also, remove the figures from your manuscript file but keep the legends.

For more information about figure files please see our guidelines:

<https://journals.plos.org/digitalhealth/s/figures>
<https://journals.plos.org/digitalhealth/s/figures#loc-file-requirements>

This has been addressed.

2. We have noticed that you have uploaded Supporting Information files, but you have not included a **list of legends**. Please add a full list of legends for your Supporting Information files **after the references list.**

This has been addressed.

3. As required by our policy on Data Availability, please ensure your manuscript or supplementary information includes the following:

A numbered table of all studies identified in the literature search, including those that were excluded from the analyses.

For every excluded study, the table should list the reason(s) for exclusion.

If any of the included studies are unpublished, include a link (URL) to the primary source or detailed information about how the content can be accessed.

A table of all data extracted from the primary research sources for the systematic review and/or meta-analysis. The table must include the following information for each study:

Name of data extractors and date of data extraction

Confirmation that the study was eligible to be included in the review.

All data extracted from each study for the reported systematic review and/or meta-analysis that would be needed to replicate your analyses.

If data or supporting information were obtained from another source (e.g. correspondence with the author of the original research article), please provide the source of data and dates on which the data/information were obtained by your research group.

If applicable for your analysis, a table showing the completed risk of bias and quality/certainty assessments for each study or outcome.  Please ensure this is provided for each domain or parameter assessed. For example, if you used the Cochrane risk-of-bias tool for randomized trials, provide answers to each of the signalling questions for each study. If you used GRADE to assess certainty of evidence, provide judgements about each of the quality of evidence factor. This should be provided for each outcome.

An explanation of how missing data were handled.

This information can be included in the main text, supplementary information, or relevant data repository. Please note that providing these underlying data is a requirement for publication in this journal, and if these data are not provided your manuscript might be rejected.

This has been addressed.

4. We note that your Data Availability Statement is currently as follows: “This systematic review contains all relevant data.”.
 
Please confirm at this time whether or not your submission contains all raw data required to replicate the results of your study. Authors must share the “minimal data set” for their submission. PLOS defines the minimal data set to consist of the data required to replicate all study findings reported in the article, as well as related metadata and methods (<https://journals.plos.org/plosone/s/data-availability#loc-minimal-data-set-definition>).
 
 For example, authors should submit the following data:
 
 - The values behind the means, standard deviations and other measures reported;
 - The values used to build graphs;
 - The points extracted from images for analysis.
 
 Authors do not need to submit their entire data set if only a portion of the data was used in the reported study.
 
 If your submission does not contain these data, please either upload them as Supporting Information files or deposit them to a stable, public repository and provide us with the relevant URLs, DOIs, or accession numbers. For a list of recommended repositories, please see <https://journals.plos.org/plosone/s/recommended-repositories>.
 
 If there are ethical or legal restrictions on sharing a de-identified data set, please explain them in detail (e.g., data contain potentially sensitive information, data are owned by a third-party organization, etc.) and who has imposed them (e.g., an ethics committee). Please also provide contact information for a data access committee, ethics committee, or other institutional body to which data requests may be sent. If data are owned by a third party, please indicate how others may request data access.

We have provided all relevant data and links to source papers

5. Some material included in your submission may be copyrighted. According to PLOS’s copyright policy, authors who use figures or other material (e.g., graphics, clipart, maps) from another author or copyright holder must demonstrate or obtain permission to publish this material under the Creative Commons Attribution 4.0 International (CC BY 4.0) License used by PLOS journals. Please closely review the details of PLOS’s copyright requirements here: PLOS Licenses and Copyright. If you need to request permissions from a copyright holder, you may use PLOS's Copyright Content Permission form.

Please respond directly to this email or email the journal office and provide any known details concerning your material's license terms and permissions required for reuse, even if you have not yet obtained copyright permissions or are unsure of your material's copyright compatibility.

Potential Copyright Issues:

We do not publish any copyright or trademark symbols that usually accompany proprietary names, eg (R), (C), or TM (e.g. next to drug or reagent names). Therefore please remove all instances of trademark/copyright symbols throughout the text, including (“Microsoft Excel Spreadsheet©”) on page 14.

No copyrighted information is included

**Review Comments to the Author

Reviewer #1:**

**Comment:** 1. In the introduction, it is not clear whether you are arguing that having children in the ER is positive or negative. I think some specificity on the appropriate times to take children to the ER would help. You should also define health literacy in your study. Line 148 mentions other factors, but only mention one specifically.

**Response:** Thank you for the comment. We have taken the time to carefully rewrite the introduction and background into one concise introduction. This is to enhance readability and address the feedback suggested by reviewers. Lines 71-135, pg 3-6.

**Comment:** There is also a bit of confusion with the continuity from the last review. While I understand the 2014 cut off, it is not clear what about 2014 marks it as the "pre-digital era". I think if there was a bit more clarity on the state of digital interventions before 2014 (as you say they are not included in the previous review) and/or other forms of interventions since 2014, that would be helpful.

**Response:** Thank you for the feedback. We have added further information, lines 131-132, pg 6. indicating this period as prior to the first-time digital health interventions as a term was introduced as term in peer reviewed research.

**Comment:** In line 163, 16.63% does not seem very compelling for your argument.

**Response:** Thank you for the feedback. We have amended this section to remove this due to the limitation of the small percentage within the revised background. Lines 71-135, pg 3-6.

**Comment:** In line 391, the word tool in MMAT tool is redundant.

**Response:** Thank you we have removed this work from the sentence. Lines 276-284. Pg, 12

**Comment:** I would re-word the sentence in lines 752-754, it is a bit confusing.

Response: We have revised this statement to offer clarity. Lines, 642-645. Pg 14.

**Reviewer #2:**

Abstract
**Comment:** 1. The abstract currently lacks a clear summary of the results. Please include key details such as the number of studies assessed and included, the number of patients, and a general summary of the main findings.

**Response:** Thank you for the feedback. The number of papers assessed is detailed in line 44. We have added the total number of parents. Due to the significant number of findings within the narrative review and restrictions of the abstract word count we are unable to provide a complete summary. Lines 37-44. Pg 2.

**Comment:** 2. The term “digital intervention” is not defined—please clarify this early in the abstract.

**Response:** We have included a definition alongside this intervention within the abstract. Lines 30-33, pg 2.

Introduction
**Comment:** 1. Overall, the introduction provides a thorough background and justification for the study. I especially appreciate the attention to global diversity in the interventions reviewed.

**Response:** Thank you for you positive feedback regarding the introduction.

**Comment:** 2. My main concern with the introduction is that it feels overly lengthy and at times repetitive. This made it a bit hard to review. Many points are restated across different paragraphs. I recommend revising to make it more concise and remove redundant information. It would also help to break up longer paragraphs (such as the first one).

**Response:** Thank you for the comment. We have taken the time to carefully rewrite the introduction and background into one concise introduction. This is to enhance readability and address the feedback suggested by reviewers. Lines 71-135, pg 3-6.

**Comment:** 3. Consider integrating the background section into the main introduction to streamline the structure, as the current introduction essentially functions as a background section.

**Response:** Thank you. We have now integrated both sections into one concise introduction. Lines 71-135, pg 3-6.

**Comment:** 4. Please define digital interventions earlier in the introduction, particularly for those unfamiliar with the term.

**Response:** Thank you. We have now integrated both sections and made this more concise. This reduces the overall word count and after defining the problem, offers an introduction to digital health interventions earlier into the abstract to inform paragraph three in the new introduction.

**Comment:** 5. It would be helpful to clearly define the research gap and the aim of the study earlier in the introduction.
**Response:** Thank you. By condensing the introduction and background, we believe this offers a more direct and streamlined route to the research gap and the review aims. Lines 71-135, pg 3-6.

Methods
**Comment:** 1. Line 256: The wording of the inclusion criteria is difficult to follow. Also, it would be clearer if the inclusion criteria were presented in Table 1. Currently, it's unclear how the text description relates to what is shown in the table.

**Response:** Thank you for the feedback. We have added the inclusion criteria into Table 1 to ensure this offers clarity to eligibility. Lines 168-169. Pg 7-8.

**Comment:** 2. The section on study selection criteria is hard to follow as the structure is unclear and a lot of information is repeated. Making this section more streamlined would improve clarity.

**Response:** Thank you for the feedback. This section has been amended for clarity and ease of reading. Lines 200-205, pg 10.

**Comment:** 3. In Figure 1, 2,936 records are shown as excluded. Please clarify the reasons for exclusion.

**Response:** We have amended the PRISMA diagram to indication the rationale for exclusion within Figure 1. Lines 250-284. Pg 11.

**Comment:** 4. Also in Figure 1, 3 reports were not retrieved. It would be helpful to provide reasons for this.

**Response:** Thank you. We have provided a rationale within the figure to justify why reports could not be retrieved. Lines 250-284. Pg 11.

**Comment:** 5. Table 2 presents data quality results, but there are many “NA” entries. Please explain why certain questions were not applicable and elaborate on why some studies did not meet the data quality standards. a. This may be addressed in the subsequent table in the Results section, but the placement is confusing. Consider consolidating or cross-referencing the two tables more clearly.

**Response:** Thank you for the feedback. We have added a line describing how the MMAT tool has five categories of study designs and N/A represents when a study design does not apply to the included paper. Lines 307-315. Pg 12. We have also added a line in the quality assessment box in Table 3 to indicate the category of study design according to the MMAT tool. This can be crossed referenced with the ‘Design’ column to improve traceability. 245-347. Pg

**Comment:** 6. I appreciate that a registered protocol is cited (Milne-Ives et al., 2021), which enhances the transparency of the review process.

**Response:** Thank you for the positive comment.

**Comment:** 7. The literature search appears thorough. To increase transparency, please provide examples of the search terms used.

**Response:** Due to the broadness of the search strategy, it is difficult to pick a few relevant terms as examples for the main text, therefore, we have moved supplementary table 1 into the main manuscript as identification of all search terms utilised in the search. Lines 197-198. Pg 9.

**Comment:** 8. For the narrative synthesis section, please elaborate on the methods used. How were themes identified and generated?
**Response:** Thank you for the feedback. We have added further content to the section on data analysis to elaborate on the methods used. Lines 329-337. Pg 14-15.

Results
**Comment:** 1. The authors present a thorough review of the literature, and the focus on key concepts like accessibility is appreciated.

**Response:** Thank you for the positive feedback regarding the results section.

Discussion
**Comment:** 1. Like the introduction, the discussion is quite long and contains redundant information, which makes it difficult to follow/review. While there is valuable detail, the section would benefit from being more concise.

**Response:** thank you for the feedback. We have reviewed and revised the discussion section to make this more concise. Lines 667-792. Pg 14-18.

**Comment:** a. Several paragraphs are overly long and could be broken up for better readability.
**Response:** Thank you for the feedback. We have further condensed the discussion and amended the paragraph length to improve overall readability. Lines 667-792. Pg 14-18.

General
**Comment:** 2. In general, there are a number of grammatical errors throughout the manuscript (many in intro and methods). I recommend the authors review and edit for grammar, clarity, and language quality to improve readability.

**Response:** Thank you for the feedback. We have conducted further reading of the review to ensure that any potential grammatical errors have been addressed, including the language quality.

**Reviewer #3:**

**Comment:** A very thoughtful topic as per the rise in digitalization era, but there are some modifications required before the final acceptance of the manuscript. The reference list mentioned is in a haphazard manner, so kindly arrange the reference list properly with adequate spacing.

Response: Thank you for the comment. We have adjusted the reference list and ensured consistency in reporting.
